# Supplementary material for: Risk factors for sacrococcygeal pilonidal sinus: a systematic review and meta-analysis supplemented by genetic causal assessment
Source: Front Surg. 2026 Jan 7;12:1718589. doi: 10.3389/fsurg.2025.1718589 (PMC12819706; doi:10.3389/fsurg.2025.1718589)
Supplement: Supplementary file 2 [file Datasheet2.zip › Supplementary Data 2/MR_pipeline_p5e-8/leaveone_plots/ukb-saige-272.11.finngen_R12_L12_PILONIDALCYST.pdf]

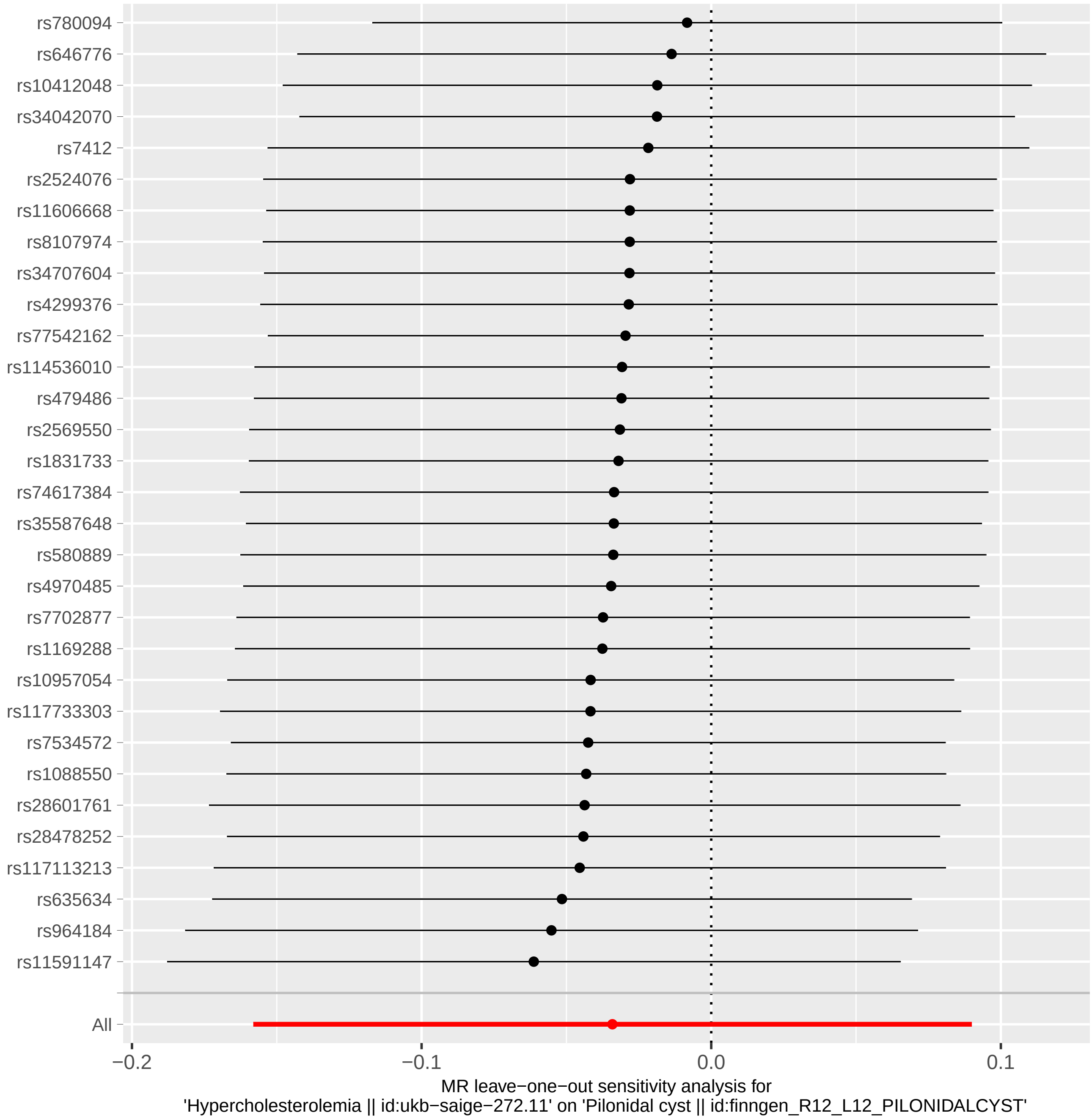
rs780094
rs646776
rs10412048
rs34042070
rs7412
rs2524076
rs11606668
rs8107974
rs34707604
rs4299376
rs77542162
rs114536010
rs479486
rs2569550
rs1831733
rs74617384
rs35587648
rs580889
rs4970485
rs7702877
rs1169288
rs10957054
rs117733303
rs7534572
rs1088550
rs28601761
rs28478252
rs117113213
rs635634
rs964184
rs11591147
All
−0.2
−0.1
0.0
0.1
MR leave−one−out sensitivity analysis for
'Hypercholesterolemia || id:ukb−saige−272.11' on 'Pilonidal cyst || id:finngen_R12_L12_PILONIDALCYST'
